# Seroprevalence and Risk Factors for *Coxiella burnetii* (Q Fever) Seropositivity in Dairy Goat Farmers' Households in The Netherlands, 2009–2010

**DOI:** 10.1371/journal.pone.0042364

**Published:** 2012-07-27

**Authors:** Barbara Schimmer, Anke Lenferink, Peter Schneeberger, Helen Aangenend, Piet Vellema, Jeannine Hautvast, Yvonne van Duynhoven

**Affiliations:** 1 Centre for Infectious Disease Control, National Institute for Public Health and The Environment, Bilthoven, The Netherlands; 2 Academic Collaborative Centre AMPHI, Department of Primary and Community Care, Radboud University Nijmegen Medical Centre, Nijmegen, The Netherlands; 3 Department of Medical Microbiology and Infection Control, Jeroen Bosch Hospital, 's-Hertogenbosch, The Netherlands; 4 Department of Small Ruminant Health, Animal Health Service, Deventer, The Netherlands; University of São Paulo, Brazil

## Abstract

Community Q fever epidemics occurred in the Netherlands in 2007–2009, with dairy goat and dairy sheep farms as the implicated source. The aim of the study was to determine the seroprevalence and risk factors for seropositivity in dairy goat farmers and their household members living or working on these farms. Sera of 268 people living or working on 111 dairy goat farms were tested for *Coxiella burnetii* IgG and IgM antibodies using immunofluorescence assay. Seroprevalences in farmers, spouses and children (12–17 years) were 73.5%, 66.7%, and 57.1%, respectively. Risk factors for seropositivity were: performing three or more daily goat-related tasks, farm location in the two southern provinces of the country, proximity to bulk milk-positive farms, distance from the nearest stable to residence of 10 meters or less, presence of cats and multiple goat breeds in the stable, covering stable air spaces and staff not wearing farm boots. Goat farmers have a high risk to acquire this occupational infection. Clinicians should consider Q fever in this population presenting with compatible symptoms to allow timely diagnosis and treatment to prevent severe sequelae. Based on the risk factors identified, strengthening general biosecurity measures is recommended such as consistently wearing boots and protective clothing by farm staff to avoid indirect transmission and avoiding access of companion animals in the goat stable. Furthermore, it provides an evidence base for continuation of the current vaccination policy for small ruminants, preventing spread from contaminated farms to other farms in the vicinity. Finally, vaccination of seronegative farmers and household members could be considered.

## Introduction

Q fever is a ubiquitous zoonosis caused by the bacterium *Coxiella burnetii (C. burnetii)*. The primary animal reservoirs for human infection are cattle, sheep and goats. Bacteria are shed in especially high concentrations in placentas and birth fluids of infected animals, which may subsequently contaminate the stable environment [1]. Human infection results from inhalation of contaminated aerosols, generated by infected animals or animal products or through direct contact with milk, urine, faeces, or semen of infected animals [2], [3], [4]. Human Q fever may range from subclinical infection to endocarditis and ruptured aneurysms, and long-term sequelae such as chronic fatigue syndrome *(1–4)*. Ruminant farmers are considered one of the main occupational risk populations for acquiring *C. burnetii* infections [5]. In 2009–2010, our integrated human-animal-environmental Q-VIVE study among Dutch dairy goat farms showed a farm prevalence of 43.1% and a goat seroprevalence of 21.4% [6]. The aim of the present study was to determine within the same farm study population, the seroprevalence in farmers and household members living and/or working on dairy goat farms and to assess the farm-related and individual risk factors for seropositivity in order to update control measures and to provide targeted advice for this occupational group and the Dutch dairy goat industry.

## Methods

All dairy goat farms in the Netherlands with at least 100 adult goats that were not vaccinated for Q-fever were selected from a national database of the Animal Health Service. On eligible farms, we approached dairy goat farmers and one or two of their household members aged 12 years and older, and in some instances, other persons working or living on the farm such as farm employees. A maximum of three participants were included per farm. Non-responders received a reminder three weeks after the initial invitation. After providing informed consent on farm and individual level, all participating farms were visited by professional laboratory assistants, who collected sera from October 2009 through March 2010. Each participant received an individual questionnaire by e-mail or post containing questions on person-based exposures, for instance living and/or working on the farm, contact with goats, other livestock, pets and the farm environment, consumption of raw dairy products, use of protective clothing, pregnancy, smoking and underlying health conditions. A farm questionnaire was sent to the farm manager/owner containing questions on herd size, presence of other livestock and pets, farm management, stable environment, lambing season and hygiene measures. We obtained data on the Q fever bulk milk status for the period 1 October 2009–30 September 2010 from the Dutch Ministry of Agriculture. The Medical Ethical Commission of the University Medical Center Utrecht approved the study protocol (nr. 09–189/K).

### Serology

Serum samples were tested for *C. burnetii* IgM and IgG antibodies, both phase I and II, using indirect immunofluorescence assay (IFA) with a screening dilution of 1∶32. Study participants without any positive antibody result and participants with a solitary IgM phase I or solitary IgM phase II were defined as seronegative. All other outcomes were classified as seropositive. Those with among others IgM phase II antibodies were designated as ‘relatively recent infections’ and include possible current infections. The term ‘relatively recent’ was chosen as IgM phase II is found positive (≥1∶32) in the majority of cases one year post-infection and may even persist up to three years post-infection [7], (personal communication C. Wielders). Seropositives without IgM phase II antibodies were designated as ‘past infections’. As the latter also includes possible chronic infections, within the past infections a distinction was made between serological profiles which had IgG phase I <1∶32 or negative and therefore not consistent with chronic infection, and serological profiles which could indicate chronic infection. IgG phase I and II end point titers were determined.

### Data analysis

To study participation bias, participating and non-participating farms were compared with regard to herd size, urbanization degree, region and bulk milk status. For the risk factor analyses, first frequency tables of variables were analysed and distributions of continuous variables were studied. If the latter were not linearly related to the outcome variable, variables were divided into classes based on biological arguments and if these were lacking based on quartiles or medians and/or chosen similarly to classes used in a previous analyses of risk factors for the goats of these same farms [6]. Using SAS software version 9.2, univariate logistic regression analysis was performed to assess the main factors associated with *C. burnetii* seropositivity at the individual level (p<0.20 in the likelihood ratio test (−2LL)). Variables with less than 10% of data in one risk category were excluded. Age was always kept in the model because of the frequent relationship with *C. burnetii* seropositivity observed in other studies. Proxy outcomes such as bulk milk Q fever status were not included in multivariable analyses. All identified individual variables were analyzed with a manual backward elimination procedure starting with a full multivariable logistic regression model. Variables were kept in de model if the −2LL ratio test of the model with and without the variable was significant (p<0.05). The final individual model was tested with the Hosmer-Lemeshow-Goodness-of-fit test. Subsequently, multilevel univariate model analyses were performed to identify risk factors derived from the farm questionnaire, taking into account clustered farm-based data for all persons within the same farm, using a unique farm number as cluster variable for each farm. All univariately significant farm variables (p<0.20) were analyzed with a manual backward elimination procedure starting with a full multilevel model. Finally, both the individual and farm-based characteristics from the final two submodels were combined in a multivariate multilevel analysis, to examine the independent relationship between risk determinants and *C. burnetii* seropositivity. The final model fit was assessed by the QIC (Quasi-Likelihood under the Independence Model Criterion) goodness-of -fit statistic for GEE (generalized estimation equation) models.

## Results

### Descriptive characteristics

Of all 334 invited eligible dairy goat farms, 111 (33.2%) farms participated in this study. In total, 24.3% of the participating farms tested bulk milk-positive from October 2009 through March 2010, similar to 22.9% positive for the non-participating farms. The mean herd size was 869 goats (range 121–3805) in participating farms, not statistically different from the mean of 809 goats (105–4733) in non-participating farms. In addition, no differences between participating and non-participating farms were observed with regard to urbanization degree and region. From the 111 participating farms, 268 persons provided a blood sample (mean age 42.0 years (12–81), 53.7% male). Of these, 184 (68.7%) were seropositive; 154 (57.5%) participants had experienced a past infection and 30 (11.2%) had experienced a relatively recent infection, as demonstrated by presence of IgM phase II antibodies. IgG phase II end titres were known for the 75 participants with a past infection with IgG phase I <1∶32: 1∶32 (n = 20), 1∶64 (n = 14), 1∶128 (n = 16), 1∶256 (n = 12), 1∶512 (n = 9) and ≥1∶1024 (n = 4). For the 79 participants with a past infection with IgG phase I ≥1∶32, 11 persons had ‘possible chronic Q fever’ with IgG phase I titers ≥1∶1024 according to diagnostic criteria used in the Netherlands [8]. Clinical information was lacking to confirm that these were truly chronic cases. Based on questions regarding clinical history in the individual questionnaire, none of them reported a history of pneumonia, hepatitis or endocarditis during the past 5 years or were diagnosed with acute Q fever by their general practitioner. Also none of them indicated a history of an immune disorder, chronic pulmonary disease or cardiovascular problem except for one case with a high blood pressure and one case with a breast malignancy in 1999. Seroprevalences in males increased by age reaching a plateau around 80% in the 35–55 years age group, while in females the highest seroprevalence (75.9%) was observed in those below 35 years. The seroprevalence was highest among farmers (73.5%) and in the small group of non-household members such as farm employees or servants (83.3%). Seroprevalences in spouses and children (12–17 years) were 66.7% and 57.1%, respectively (Table 1). Among those living or working on a bulk milk-positive farm, 95.5% were seropositive. The median duration of residence on a dairy goat farm was 10 years (0–29 years). A Q fever episode was confirmed by a general practitioner in 10 participants (4.1%) during the period 2008–2010. Based on their serum sample, 5 had a serological profile matching relatively recent infection and 5 a profile indicating past infection.

**Table 1:** Descriptive characteristics and seroprevalence of dairy goat farmers and family members (n = 268), September 2009–April 2010, The Netherlands.

Variable	Frequency (N)	Seroprevalence	
		% (N)	95% CI (exact)
Participant	268	68.7 (184)	62.7–74.2
Sex			
Male	144	70.1 (101)	62.0–77.5
Female	124	66.9 (83)	57.9–75.1
Age (years) in males			
<35	36	55.6 (20)	38.1–72.1
35-<45	43	79.1 (34)	64.0–90.0
45-<55	35	80.0 (28)	63.1–91.6
≥55	30	63.3 (19)	43.9–80.1
Age (years) in females			
<35	29	75.9 (22)	56.5–89.7
35-<45	47	61.7 (29)	46.4–75.5
45-<55	31	67.7 (21)	48.6–83.3
≥55	17	64.7 (11)	38.3–85.8
Function			
Farmer	132	73.5 (97)	65.1–80.8
Spouse	69	66.7 (46)	54.3–77.6
Farmers' son/daughter ≥18 years	21	66.7 (14)	43.0–85.4
Child (12–17 years)	14	57.1 (8)	28.9–82.3
Other family members	26	53.9 (14)	33.4–73.4
Other persons, such as farm employees	6	83.3 (5)	35.9–99.6

*CI, Confidence Interval.*

### Univariate analyses on individual and farm level

Individual farm exposures, such as milking and feeding goats, supply and removal of dairy goats into and outside the stable, giving general health care and birth assistance, cleaning the stables, removal and spread of manure, contact with raw goat milk and daily contact with goat manure, dead-born animals during the lambing season, daily contact with roughage or animal feed, direct contact with cattle on own farm and residence as a child on a ruminant farm were all associated with human seropositivity (p<0.05). Potential risk factors with p≥0.05 to p<0.20 were having lived and/or worked before 1997 on a dairy goat farm, being a farmer, fulltime work week, having a dog, direct contact with horses on own farm, daily contact with birth material during the lambing season, contact with cattle manure and contact with goats, dogs and cats at other farms and work experience in the cattle sector (Table 2).

**Table 2:** Univariate logistic model of individual factors associated with human Q-fever (P<0.20) with corresponding frequency (N), seroprevalence (%), odds ratio (OR) and 95% confidence interval (CI).

Variable	Category	Freq. (N)	Seropre-valence (%)	OR [95% CI]	p-value
Age group	<35 years	65	64.6	Reference	0.57
	35-<45 years	90	70.0	1.28 [0.65–2.52	
	45-<55 years	66	74.2	1.58 [0.75–3.34]	
	≥55 years	47	63.8	0.97 [0.44–2.11]	
Start working on goat farm	1997 or after	141	66.0	Reference	0.07
	before 1997	107	76.6	1.69 [0.96–2.99]	
Function	Farmer	122	74.6	1.54 [0.89–2.68]	0.13
	Other	122	65.6	Reference	
Hours working on farm	Fulltime	124	76.6	2.46 [1.25–4.82]	0.05
	Halftime	35	74.3	2.17 [0.86–5.46]	
	Quarter week	29	62.1	1.23 [0.49–3.07]	
	Sometimes/never	56	57.1	Reference	
Number of daily goat-related tasks (milking, feeding, supply and removal, general animal health care, birth assistance)	≥3 tasks	167	79.6	3.69 [2.14–6.34]	<0.01
	≤2 tasks	101	50.5	Reference	
Milking goats	Yes	183	75.4	2.60 [1.42–4.77]	<0.01
	No	61	54.1	Reference	
Feeding goats	Yes	186	76.3	3.23 [1.74–5.97]	<0.01
	No	58	50.0	Reference	
Supply and removal of dairy goats or bucks	Yes	120	82.5	3.41 [1.89–6.15]	<0.01
	No	124	58.1	Reference	
Care for general animal health	Yes	151	78.8	2.93 [1.67–5.16]	<0.01
	No	93	55.9	Reference	
Birth assistance	Yes	151	78.2	2.70 [1.54–4.74]	<0.01
	No	93	57.0	Reference	
Remove manure	Yes	130	76.9	2.02 [1.16–3.52]	0.01
	No	114	62.3	Reference	
Spread manure	Yes	69	82.6	2.54 [1.27–5.10]	<0.01
	No	175	65.1	Reference	
Clean the stables	Yes	118	81.4	2.97 [1.66–5.32]	<0.01
	No	126	59.5	Reference	
Administration	Yes	148	74.3	1.66 [0.95–2.90]	0.07
	No	96	63.5	Reference	
Having a dog	Yes	209	71.8	1.70 [0.81–3.55]	0.16
	No	35	60.0	Reference	
Direct contact cattle own farm	Yes	34	85.3	2.78 [1.03–7.55]	0.04
	No	210	67.6	Reference	
Direct contact horses own farm	Yes	90	76.7	1.68 [0.93–3.03]	0.09
	No	154	66.2	Reference	
Direct contact goats other farm	Yes	28	82.1	2.11 [0.77–5.80]	0.15
	No	216	68.5	Reference	
Direct contact horses other farm	Yes	24	83.3	2.29 [0.75–6.94]	0.14
	No	250	68.6	Reference	
Direct contact dogs other farm	Yes	81	77.8	1.78 [0.96–3.30]	0.07
	No	163	66.3	Reference	
Direct contact cats other farm	Yes	29	82.8	2.22 [0.81–6.07]	0.12
	No	215	68.4	Reference	
Daily contact roughage/animal feed	Yes	174	74.1	1.91 [1.06–3.44]	0.03
	No	70	60.0	Reference	
Contact raw milk	Yes	182	75.3	2.51 [1.37–4.58]	<0.01
	No	62	54.8	Reference	
Daily contact goat manure	Yes	163	75.5	2.11 [1.20–3.74]	0.01
	No/no respons	81	59.3	Reference	
Contact cattle manure	Yes	32	84.4	2.55 [0.94–6.91]	0.07
	No	212	67.9	Reference	
Daily contact dead-born animals	Yes	100	80.0	2.33 [0.94–6.91]	<0.01
	No	143	63.2	Reference	
Daily contact with placenta/birth material	Yes	156	73.1	1.48 [0.84–2.59]	0.17
	No/no respons	88	64.8	Reference	
Work in agriculture (swine)	Yes	79	77.2	1.69 [0.91–3.14]	0.09
	No	165	66.7	Reference	
Work in agriculture (cattle)	Yes	134\	74.6\	1.62 [0.92–2.80]	0.09
	No	110	64.5	Reference	
Work in agriculture (crops)	Yes	53	77.4	1.60 [0.79–3.27]	0.19
	No	191	68.1	Reference	
Work in transport (agricultural products, such as hay, straw)	Yes	31	83.9	2.44 [0.90–6.63]	0.08
	No	213	68.1	Reference	
Lived as child on a ruminant farm	Yes	172	74.4	1.79 [0.96–3.32]	0.02
	No	72	59.7	Reference	

The following farm-based characteristics were potential risk factors in univariate analyses: farm location (south), short distance to the nearest bulk milk-positive farm, bulk milk status from 1 October 2009 to 30 September 2010, herd size ≥800 goats, distance between residence and nearest goat stable ≤10 meters, presence of other goat breeds besides the Dutch White Goat, use of artificial insemination, extended lactation, presence of cats in the stable, use of silage and/or maize feed, using a fodder mixer or automatic feeding method, use of screen/gauze in the stable, covering air spaces in the stable for combating nuisance animals such as wild birds, ≥3 lambing periods per year, an abortion percentage of ≥4% in 2007–2009 or a reported Q fever abortion wave. Biosecurity factors such as having a closed farm tenure, goat supply from southern provinces, receiving farm visitors for school tours or organised groups, and not wearing farm boots among staff and other employees were also potential risk factors. Two spatial variables were potential risk factors: municipal cattle density of 100 ruminants per km^2^ and a net goat density of ≥15 goats per km^2^ within five kilometre radius. Besides risk factors, potential protective factors were found, such as presence of laying hens, stable air ventilation through a flap, membership of an organic dairy goat cooperative, and presence of rats or mice in the stable in 2008 before implementation of the hygiene protocol (Table 3).

**Table 3:** Univariate multilevel model of farm-based factors associated with human Q-fever (p<0.20) with corresponding frequency (N), seroprevalence (%), odds ratio (OR) and 95% confidence interval (CI).

Variable	Category	Freq. (N)	Seropre-valence (%)	OR [95% CI]	p-value
Herd size	≥800 goats	124	75.8	1.82 [1.07–3.11]	0.03
	<800 goats	144	63.2	Reference	
Farm region	North (Dr, Fr, Gro)	39	51.3	Reference	<0.01
	East (Ge, Ov, Fle)	92	67.4	1.96 [0.91–4.22]	
	West (Ut, Ze, Nh, Zh)	37	56.8	1.25 [0.51–3.08]	
	South (Nb, Li)	100	82.0	4.33 [1.93–9.72]	
Q fever mandatory vaccination area 2009	Yes	124	83.1	3.71 [2.09–6.58]	<0.01
	No	144	56.9	Reference	
Bulk milk farm status	Positive	67	95.5	14.1 [4.28–46.45]	<0.01
	Negative	201	60.2	Reference	
Serological farm status (≥1 goat seropositive)	Positive	100	93.0	12.1 [5.21–28.10]	<0.01
	Negative	128	52.3	Reference	
Net goat density per km^2^ within 5 km radius	≥15	110	79.1	2.32 [1.32–4.06]	<0.01
	<15	158	62.0	Reference	
Cattle density (incl meat calves)	≥100	183	72.1	1.42 [0.81–2.47]	0.13
	<100	82	64.6	Reference	
Bulk milk-positive farm within 5 km radius	Yes	81	81.5	2.51 [1.33–4.74]	<0.01
	No	187	63.6	Reference	
Distance to nearest positive farm	0- <4 km	74	83.8	4.74 [2.18–10.31]	<0.01
	4- <8 km	50	76.0	2.90 [1.30–6.48]	
	8- <16 km	75	65.3	1.73 [0.88–3.38]	
	≥16 km	69	52.2	Reference	
Farm part of organic goat farming cooperation	Yes	38	57.9	0.57[0.28–1.14]	0.12
	No	220	70.9	Reference	
Distance residence to nearest stable	≤10 m	118	77.1	2.06 [1.17–3.61]	0.01
	>10 m	124	62.1	Reference	
Other goat breeds next to white dairy goat	Present	125	78.4	2.22 [1.27–3.88]	<0.01
	Absent	124	62.1	Reference	
Number of stables	>3 stables	55	78.2	1.68[0.83–3.41]	0.15
	≤3 stables	194	68.0	Reference	
Use of artificial insemination	Yes	62	80.7	2.05[1.02–4.13]	0.04
	No	185	67.0	Reference	
Extended lactation	Yes	212	72.6	Reference	0.03
	No	32	53.1	0.43 [0.20–0.91]	
Laying hens on farm	Yes	40	55.0	0.45 [0.22–0.90]	0.03
	No	209	73.2	Reference	
Presence of cat(s) in goat stable	Present	91	75.8	1.54 [0.86–2.76]	0.15
	Absent	158	67.1	Reference	
Use of silage	Yes	165	75.8	2.13 [1.21–3.73]	<0.01
	No	84	59.5	Reference	
Use of maize	Yes	95	81.1	2.44[1.33–4.50]	<0.01
	No	154	63.6	Reference	
Feeding method	Fodder mixer or automatic	159	74.8	1.81[1.04–3.15]	0.04
	Hand/wheelbarrow	90	62.2	Reference	
Use of screen/gauze	Screen/gauze	91	78.0	1.86 [0.91–3.80]	0.12
	Windstoppers only	94	66.0	1.01 [0.52–1.98]	
	None of the above	64	65.6	Reference	
Air ventilation through flap	Yes	33	57.6	0.52 [0.25–1.11]	0.10
	No	216	72.2	Reference	
Presence of Mice and/or rats in the stable in 2008	Present	171	67.3	Reference	0.09
	Absent	76	77.6	1.69 [0.90–3.16]	
Combat other nuisance animals in 2008	Via covering airspaces	32	84.4	2.52[0.93–6.82]	0.05
	Not via covering airspaces or no combat	217	68.2	Reference	
Lambing periods	≥3 or more periods	21	90.5	4.39 [1.00–19.33]	0.05
	≤2 lambing periods	228	68.4	Reference	
Abortion percentage 2007–2009 including abortion history due to Q fever	>4 percent	36	86.1	2.97 [1.11–7.97]	0.02
	0–4 percent	213	67.6	Reference	
Farm tenure	Closed farm for male and female goats	63	77.8	1.68 [0.86–3.28]	0.12
	Not completely closed	185	67.6	Reference	
Goat supply region	South	77	83.1	1.68 [1.87–7.69]	<0.01
	Other regions	108	56.5	Reference	
	No supply	64	78.1	2.75[1.36–5.56]	
Farm visitors	School tours	98	75.5	1.53 [0.86–2.70]	0.14
	No tours	151	66.9	Reference	
Farm boots for staff	Yes	164	66.5	Reference	0.03
	No	82	79.3	1.93 [1.03–3.60]	

### Multivariate and multilevel analyses

Three individual and eight farm-based variables were significant in the two final multivariate submodels (p<0.05, −2LL) (Tables 4 and 5, respectively) and together with age group used as the full multilevel model. The final combined individual-farm multilevel model showed that the number of daily performed goat-related tasks (including milking, feeding, supply and removal of goats, general health care, birth assistance of goats) was an independent significant risk factor as well as farm location in the two southern provinces, distance to nearest bulk milk-positive farm, presence of a cat in the goat stable, distance between residence and goat stable ≤10 meters, presence of other goat breeds besides the Dutch White Goat and not wearing farm boots among staff and other employees (Table 6). A borderline significant risk factor and a protective factor were found, i.e. living as child on a ruminant farm and no use of extended lactation, respectively.

**Table 4:** Results of the multivariate logistic regression with individual characteristics (p<0.05, −2LL), as independent factors related to human Q-fever status.

Individual Variables	Category	OR [95% CI]	p-value
Age group	<35 years	Reference	0.867
	35-<45 years	1.04 [0.46–2.34]	
	45-<55 years	1.31 [0.54–3.19]	
	≥55 years	0.88 [0.36–2.14]	
Number of daily goat-related tasks (milking, feeding, supply and removal, general animal health care, birth assistance)	≥3 tasks	3.73 [2.00–6.94]	<.0001
	≤2 tasks	Reference	
Lived as child on a ruminant farm	Yes	2.24 [1.17–4.28]	0.015
	No	Reference	
Direct contact with cattle	Yes	2.65 [0.93–7.53]	0.068
	No/no respons	Reference	

Multivariate logistic regression model with individual characteristics * Number of observations used: 244.

**Table 5:** Results of the multilevel analyses with farm-based characteristics (p<0.05, −2LL), as independent factors related to human Q-fever status.

Farm Variables	Category	OR [95% CI]	p-value
Farm region	North (provinces Dr, Fr, Gro)	Reference	0.011
	West (provinces Ut, Ze, Nh, Zh)	0.85 [0.26–2.71]	
	East (provinces Ge, Ov, Fle)	2.26 [0.88–5.84]	
	South (provinces Nb, Li)	5.95 [2.08–16.99]	
Distance to nearest positive farm	0- <4 km	3.38 [1.25–9.11]	0.050
	4- <8 km	2.64 [1.03–6.75]	
	8- <16 km	3.46 [1.44–8.30]	
	≥16 km	Reference	
Presence of cat(s) in goat stable	Present	2.54 [1.24–5.21]	0.011
	Absent	Reference	
Distance residence to nearest stable	≤10 m	2.44 [1.27–4.67]	0.011
	>10 m	Reference	
Other goat breeds next to white dairy goat	Present	3.38 [1.61–7.09]	0.003
	Absent	Reference	
Extended lactation	Yes	Reference	0.036
	No	0.37 [0.15–0.86]	
Combat other nuisance animals in 2008	Via covering airspaces	6.03 [1.77–20.61]	0.007
	Not via covering airspaces or no combat	Reference	
Farm boots for staff	Yes	Reference	0.025
	No	2.66 [1.12–6.32]	

Multivariate multilevel model with farm characteristics * Number of observations used: 234, number of levels used: 103 (7 with missing values).

**Table 6:** Results of the multilevel analyses with all individual and farm-based variables which were associated with human Q fever status (p<0.10, −2LL) taking in account clustered data of persons within a farm.

Variable	Category	OR [95% CI]	p-value
Age group	<35 years	Reference	0.804
	35-<45 years	1.52 [0.58–3.97]	
	45-<55 years	1.22 [0.43–3.45]	
	≥55 years	1.10 [0.32–3.76]	
Number of farm tasks	≥3 tasks	3.87 [1.84–8.14]	0.001
	≤2 tasks	Reference	
Farm region	North (provinces Dr, Fr, Gro)	Reference	0.035
	West (provinces Ut, Ze, Nh, Zh)	1.14 [0.29–4.55]	
	East (provinces Ge, Ov, Fle)	2.64 [0.98–7.07]	
	South (provinces Nb, Li)	4.88 [1.67–14.26]	
Distance to nearest positive farm	0- <4 km	4.17 [1.56–11.14]	0.020
	4- <8 km	4.12 [1.53–11.13]	
	8- <16 km	5.10 [1.85–14.09]	
	≥16 km	Reference	
Presence of cat(s) in goat stable	Present	2.21 [1.01–4.84]	0.039
	Absent	Reference	
Distance residence to nearest stable	≤10 m	2.06 [1.27–5.29]	0.014
	>10 m	Reference	
Other goat breeds next to white dairy goat	Present	3.30 [1.54–7.07]	0.003
	Absent	Reference	
Extended lactation	Yes	Reference	0.071
	No	0.42 [0.18–0.98]	
Lived as child on a ruminant farm	Yes	2.01 [0.92–4.40]	0.098
	No	Reference	
Combat other nuisance animals in 2008	Via covering airspaces	6.00 [1.70–21.18]	0.006
	Not via covering airspaces or no combat	Reference	
Farm boots for staff	Yes	Reference	0.043
	No	2.51 [1.07–5.85]	

UBN used as cluster variable. Number of observations used: 227. Number of levels used: 103 (8 with missing values).

## Discussion

This is the first study addressing the seroprevalence in dairy goat households in the Netherlands, and one of few risk factor studies on human *C. burnetii* infections in farm populations worldwide. It confirms that living and or working on a dairy goat farm poses a high lifetime risk for acquisition of a *C. burnetii* infection. Farmers and other household members are generally in closest proximity to infected goats and contaminated stables on farms, and therefore at highest risk for inhaling *C. burnetii*. We investigated *C. burnetii* presence in vaginal swabs, manure, surface area swabs, milk unit filters, and aerosols at 19 Dutch dairy goat farms in the environmental study component. Farms with an abortion history and positive bulk milk status displayed the highest level of *C. burnetii* DNA in, among others, aerosols and surface area swabs, indicating an elevated risk to farm households and visitors in acquiring Q fever [9]. This is supported by our finding that one quarter of all study participants resided on a bulk milk-positive farm, of which all were seropositive except for three persons on one farm which tested bulk milk-positive two months after human sera were taken. This indicates a highly effective transmission from infected animals to humans, as was already shown in workers involved in culling goats of whom 17.5% seroconverted post-cull [10].

The observed seroprevalence was not only high for the farmers (74.2%), as expected, but also among spouses (66.7%) and children of 12–17 years (57.1%), who lived and often also worked at the farm. The seroprevalence clearly exceeds the estimates of 2.4% found in the Dutch general population preceding the first epidemic season in 2006–2007 [11], the 24% in a rural area in the epicentre of the epidemic in September 2007 [12] and the 12.2% in blood donors living in the high-endemic regions in 2009–2010 [13]. The seroprevalence was also higher compared to those in other studies focusing on, non-further specified, farm populations, such as 49% among farmers from Northern Ireland [14], and 27% in a farm cohort in the United Kingdom [5], [14], but was comparable to the seroprevalences ranging from 68% through 84% among professionals intensively working with ruminants in several other studies [15], [16], [17], [18]. In general, comparison of seroprevalences is complicated because of the different study populations, diagnostic tests and cut-off values used. The study from Northern Ireland suggested that infection is mainly acquired in adolescence and early adulthood with slight further acquisition of infection in older age groups above 35 years [14]. We observed a different seroprevalence pattern in our study, with a simialr seroprevalence among males in the age group 12 to 25 years and 25 to 34 years (55.0% and 56.3%, respectively) and observed an increase to 79.1% in males of 35 to 44 years, while females below 25 years had already a higher seroprevalence (81.3%) compared to females in the 25–34 years age group (69.2%).

This study shows Q fever in dairy goat farm households is an actual occupational disease as one out of 9 participants had an indication for a relatively recent infection. Partially these were also diagnosed in routine medical practice in the past few years and thus with symptomatic manifestations. Eleven participants (4.1%) had a serological profile indicative for a chronic infection (IgG phase I titers ≥1∶1024), classified as ‘possible chronic Q-fever’ according to the recent Dutch criteria [8]. Clinical information was lacking to confirm that these were truly chronic cases: we neither had date of onset of symptoms compatible with Q-fever in the acute stage of these participants, if any, to know that these high level antibodies were found more than six months following infection, nor had we any clinical follow-up information for these patients after the test result from the study was communicated to them through their general practitioner, who took care of the regular follow-up protocol.

Several independent individual and farm-based risk factors for *C. burnetii* seropositivity were found such as performing ≥3 daily goat-related tasks, farm location in the two southern provinces, distance to the nearest bulk milk-positive farm, presence of other goat breeds besides the Dutch White Goat, covering air spaces in the goat stable to combat nuisance animals such as wild birds, ≤10 meters distance between residence and nearest stable, presence of cats in the stable and not wearing farm boots by staff. The individual risk factor of performing ≥3 daily activities involving direct contact with goats or dust-producing activities in the goat stable, such as milking, feeding, supply and removal of goats, general health care and cleaning the stable, reflects the intensity of goat and stable environment contact. Under these circumstances the risk of inhalation of contaminated aerosols is high, with a plausible increased risk for acquiring an infection. A study among British farm workers suggested that the extent of total farm animal contact seemed more important than specific animal exposure, indicating that risk of *C. burnetii* exposure is mainly related to farm environment contact [5]. Manure-related tasks did not add to the cumulative risk of performing goat-related tasks indicating transmission through manure is probably less important as shown in a *C. burnetii* survival study showing short decimal reduction times to establish effective killing of viable bacteria in goat manure piles [19]. Persons who lived as child on a ruminant farm were more often seropositive. This is a plausible risk factor for identified past infections and in agreement with a study among Dutch veterinary students where those that grew up on a ruminant farm had a higher risk of being seropositive and a dose-response relationship between seropositivity and years of farm residence was identified [20].

Two risk factors indicate the importance of airborne transmission between farms and to humans in high-incidence areas for Q fever: a farm location in two southern provinces of the Netherlands and a closer distance to the nearest bulk milk-positive farm. The concentration of intensive goat farming in the south of the country probably facilitated transmission between farms following the introduction of Q fever. Next to the farm families, the general population in these regions was severely affected with annual incidences up to 500–1500 notifications per 100,000 inhabitants. Of 2,421 notified cases in 2007–2009, 3.2% worked in the agricultural sector including stockbreeding, arable and dairy farming [21]. Effective airborne spread between farms and to farm families from infected farms in the vicinity is also supported by the observation that 38% of the participating farms within eight kilometers proximity from a bulk milk-positive farm were bulk milk-positive themselves. The farms's own bulk milk status became a strong significant risk factor if we added it to the final multilevel model, while the significance of distance to the nearest bulk milk-positive farm decreased (data not shown), which indicated that a positive bulk milk status of the own farm is the strongest predictor for human seropositivity. A previous study showed that the risk of acute Q fever gradually decreased with increasing distance from a dairy goat farm that experienced an abortion wave [22]. The same distance-response relationship was observed for goat seropositivity in the veterinary study component [6]. A distance of ≤10 meters between stable and residence as risk factor could be a sign of more intensive transmission through aerosol spread but could also be a proxy for more intense direct human-animal contact. The presence of other goat breeds, such as Toggenburg, Anglo-Nubian, Dutch Pied or Alpine Goat, besides the omnipresent Dutch White Goat was identified as risk factor. Compared to other goats, the Dutch White goat is specifically bred on their milk-producing quality. Animal-to-human transmission of *C. burnetii* may be influenced by breed diversity [23] and in cattle the Friesian breed has higher *C. burnetii* seroprevalences than other breeds [24]. However, presence of other goat breeds as a risk factor for human seropositivity was not previously described and therefore this finding cannot be satisfactorily explained. Possible mechanisms behind this risk factor need further investigation, for example through seroprevalence studies in different goat breeds. The study further indicates that not performing extended lactation may protect against human infections which could point to a selection on dairy goats for its qualities as a high-productive dairy goat, rather than on disease resistance, which can cause undesirable side-effects in for example immunological traits, as seen in other livestock [25]. Covering air spaces in a stable to combat nuisance animals such as wild birds could point at a more air-locked stable, facilitating *C. burnetii* accumulation inside the stable, which may increase human and goat exposure [26]. The observed risk for farms where staff did not wear farm boots may indicate the need for more stringent routine biosecurity procedures for household members, farm employees and visitors as indirect transmission through contaminated clothing has been described for Q fever [26]. The presence of cats in the stable was also observed as risk factor for both human and goat seropositivity [6], suggesting *C. burnetii* introduction or facilitation of spread by infected companion animals. In the veterinary study component, additionally the presence of dogs in the stable was a risk factor.

This study has some limitations. First, the response rate of 33.2% among eligible dairy goat farms was relatively low. This was probably mainly due to the stringent measures carried out during the Q fever epidemics in the Netherlands, especially the culling of pregnant goats on bulk milk-positive farms implemented late 2009, and the media attention during the period that the farms were invited for this study. As the proportion of bulk milk-positive farms was similar for participating farms and non-participating farms, and comparable with regard to herd size, urbanization degree and regional representation, we consider the observed seroprevalence and risk factors representative for all dairy goat farmers and household members in the Netherlands. Second, the exposure information collected in the farm and individual questionnaires is not necessarily related to the relevant time period for seroconversion as we do not know when the actual *C. burnetii* infection occurred in seropositive participants. This also complicated the assessment of the clinical relevance of the high seroprevalences observed. However, a relatively high percentage of relatively recent infections occurred, indicating that seroconversion in this group most likely occurred during the periods covered in the questionnaire.

To conclude, high *C. burnetii* seroprevalences indicate dairy goat farmers and household members have a substantial lifetime risk to acquire this zoonotic infection. Our study demonstrates the importance of daily goat and stable environment contact and increased risk of living on or in proximity of a bulk milk-positive farm. We recommend strengthening general biosecurity measures such as consistently wearing boots and protective clothing by farm staff to avoid indirect transmission, avoiding access of companion animals to the stable and get advice on controlling nuisance animals in the goat stables as covering air spaces seem to harbour an increased risk. Awareness among clinicians should be increased to consider Q fever in this occupational group presenting with compatible symptoms or related sequelae to allow timely diagnosis and treatment. As preventive strategies, dairy goat farmers and household members could be screened at start of goat farming or at adolescent age for children being raised at such farms and if seronegative, offered a human Q fever vaccine. This, in addition to the earlier mentioned biosecurity measures and continuation of small ruminant vaccination, both for decreasing the exposure risk for young children at the farm not yet suitable for vaccination and for inhabitants in the vicinity of the farms.
